# Navigator channel adaptation to reconstruct three dimensional heart volumes from two dimensional radiotherapy planning data

**DOI:** 10.1186/1756-6649-12-1

**Published:** 2012-01-18

**Authors:** Angela Ng, Thao-Nguyen Nguyen, Joanne L Moseley, David C Hodgson, Michael B Sharpe, Kristy K Brock

**Affiliations:** 1 Radiation Medicine Program, Princess Margaret Hospital, Toronto, Ontario, Canada; 2 Department of Medical Biophysics, University of Toronto, Toronto, Ontario, Canada; 3 Department of Radiation Oncology, University of Toronto, Toronto, Ontario, Canada

## Abstract

**Background:**

Biologically-based models that utilize 3D radiation dosimetry data to estimate the risk of late cardiac effects could have significant utility for planning radiotherapy in young patients. A major challenge arises from having only 2D treatment planning data for patients with long-term follow-up. In this study, we evaluate the accuracy of an advanced deformable image registration (DIR) and navigator channels (NC) adaptation technique to reconstruct 3D heart volumes from 2D radiotherapy planning images for Hodgkin's Lymphoma (HL) patients.

**Methods:**

Planning CT images were obtained for 50 HL patients who underwent mediastinal radiotherapy. Twelve image sets (6 male, 6 female) were used to construct a male and a female population heart model, which was registered to 23 HL "Reference" patients' CT images using a DIR algorithm, MORFEUS. This generated a series of population-to-Reference patient specific 3D deformation maps. The technique was independently tested on 15 additional "Test" patients by reconstructing their 3D heart volumes using 2D digitally reconstructed radiographs (DRR). The technique involved: 1) identifying a matching Reference patient for each Test patient using thorax measurements, 2) placement of six NCs on matching Reference and Test patients' DRRs to capture differences in significant heart curvatures, 3) adapting the population-to-Reference patient-specific deformation maps to generate population-to-Test patient-specific deformation maps using linear and bilinear interpolation methods, 4) applying population-to-Test patient specific deformation to the population model to reconstruct Test-patient specific 3D heart models. The percentage volume overlap between the NC-adapted reconstruction and actual Test patient's true heart volume was calculated using the Dice coefficient.

**Results:**

The average Dice coefficient expressed as a percentage between the NC-adapted and actual Test model was 89.4 ± 2.8%. The modified NC adaptation technique made significant improvements to the population deformation heart models (p = 0.01). As standard evaluation, the residual Dice error after adaptation was comparable to the volumetric differences observed in free-breathing heart volumes (*p = 0.62*).

**Conclusions:**

The reconstruction technique described generates accurate 3D heart models from limited 2D planning data. This development could potentially be used to retrospectively calculate delivered dose to the heart for historically treated patients and thereby provide a better understanding of late radiation-related cardiac effects.

## Background

Radiation-induced cardiac toxicity is a significant cause of morbidity, following treatment of Hodgkin's Lymphoma (HL) [1]. Laboratory and autopsy studies have demonstrated that radiation therapy (RT) produces a range of adverse cardiac effects, including atherosclerosis, cardiomyopathy, and valvular damage [2,3]. Several studies have investigated the relationship between late cardiac effects and radiation dose and/or radiated volume [4-7]. Emami et al. attributed the tolerance doses of cardiac tissues to therapeutic irradiation, but limited to only one type of radiation injury, pericarditis [4]. Similarly, Stewart and Fajardo reported a steep dose response (pericarditis) in HL patients with an RT field that included > 50% of the external heart contour [5]. In Hancock et al. an increased risk of coronary artery and cardiac diseases was reported when mediastinal radiation reached 40-45 Gy [6]. Furthermore, existing normal-tissue complication probability models have been reported limiting due to the assumption that all regions of the organ at risk are of equal radiosensitivity [7]. Therefore, a refinement of existing RT-related 3D dose-risk model of cardiac toxicity could potentially allow a better understanding of cardiac radiation tolerances, risk estimation of late cardiac effects, and optimization of RT treatment to minimize cardiac toxicity. However, due to the delayed onset of cardiac effects, the major barrier to the application of biophysical models remains the absence of 3D dosimetry on historically treated patients, for whom late toxicity outcomes are available, but were treated with a 2D treatment planning system. In order to obtain corresponding dose-volume and reliable late toxicity data, a 3D model reconstruction system is needed to extract 3D volume information from historical 2D planning datasets.

Various techniques have been described to reconstruct 3D organ volumes from 2D imaging data for the planning of RT and surgery [8-12]. The finite-element based deformable image registration system (FEM-DIR) has been utilized for 3D estimation of cardiac deformation [8], changes in brain interstitial pressure [9], and temporal changes in liver and lung shape due to breathing motion [10-12]. In particular, a recent development of the Navigator Channels (NC) has been used in conjunction with a biomechanical-based FEM-DIR platform, MORFEUS, to predict patient specific liver movement by adapting liver motion detected from coronal 2D images to a population liver motion model [13,14]. This method can quickly associate 1D information obtained from 2D images with 3D organ information, potentially reproducing patient-specific 3D reconstructions of a given organ. In a previous investigation, single NCs were applied on 2D digitally reconstructed radiographs (DRRs) to capture differences in patients' lung structures [12]. A linear adaptation approach was then used to accurately reconstruct patient-specific 3D lung volumes based on the structural differences [12]. The current investigation builds on this technique, using an advanced NC-adaptation technique based on a series of NCs, and a combination of linear and bilinear adaptation approaches to reconstruct cardiac volumes directly from 2D images. This technique offers the potential to retrospectively correlate 3D dosimetry results with delayed cardiac toxicities for historically treated patients. Having a better understanding of dose-volume associations could in turn provide valuable insights into estimating late cardiac toxicities, and improve future HL radiation treatments.

## Methods

### A. Overview

The advanced NC adaptation technique combines the use of a biomechanical model-based deformable image registration system with multiple NCs to generate 3D heart volumes from 2D radiotherapy planning images. This technique was tested on 15 HL patients (Test patients) whose mediastinal RT was planned with a 3D CT-based treatment planning system. This technique expands on the previous lung reconstruction technique by utilizing multiple NCs on the right and left side of the heart (vs. single NCs for lung reconstruction) to delineate the deforming curvatures of the organ. While the heart is confined within the thoracic cage, its motion within its immediate boundaries (mediastinal pleura, adjacent to the lungs) is more variable than the lungs, which is more rigidly confined within the parietal pleura adjacent to the thoracic musculoskeletal wall. Therefore, in order to capture variability in heart shape, multiple NCs are used in this study to account for curvature differences at the circumflexes of the heart. Two-dimensional digitally reconstructed radiographs (DRRs), generated from 3D CT images, were used to simulate 2D fluoroscopic planning images available from historical 2D treatment plans.

**Figure 1:**
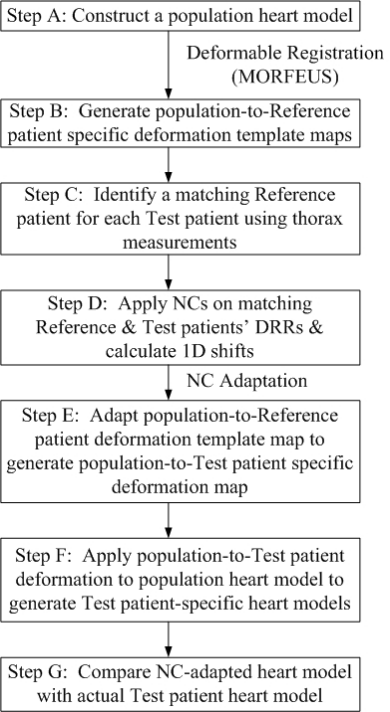
**Reconstruction process of 3D Heart Models from 2D Images**.

An overview of the technique is shown in Figure 1. To describe the reconstruction process briefly, a population heart model was first generated (Step A). The population heart model was deformed to a series of Reference patients' CT images to compile a collection of population-to-Reference patient 3D deformation template maps (Step B). A Reference patient was then selected for each Test patient based on similar thorax measurements taken on their DRRs (Step C). A series of rectangular regions of interest or NCs were applied on the comparing Reference and Test patients' DRRs to quantify patient-specific heart structures based on differences in image intensity (Step D). This patient-specific structural information was then adapted to the 3D population-to-Reference patient-specific deformation template using a combination of linear and bilinear interpolation methods to generate 3D population-to-Test patient specific deformation maps (Step E). Each population-to-Test patient deformation was then applied to the original population-to-Reference patient 3D model to recreate the Test patient's 3D heart volume (Step F). Information gained from original 3D CT images of each Test patient was used for independent evaluation of the reconstruction technique (Step G). This investigation has been approved by the University Health Network Research Ethics Board.

### B. Patient Data

A total of 50 sets of thoracic CT images from recently treated HL patients were obtained. Twelve image sets (6 male and 6 female Base patients) served as the basis for constructing the population heart models, 23 sets (Reference patients) were used to generate population-to-Reference patient deformation templates, and 15 additional sets (Test patients) were used to test the accuracy of the adaptation technique. Test patients that were treated with 3D treatment planning system between 1999 and 2008 were selected consecutively to avoid possible bias. This was then followed by consecutive selection of Reference patients that were also treated during this time period. The CT images were acquired at 120 kVp, 400 mA, with a voxel size of 0.1 × 0.1 × 0.3 cm. The patient was imaged with arms on hips, supine in an immobilization bag, under free breathing conditions. The acquired images were imported into a 3D CT-based treatment planning system (PINNACLE^3 ^v7.6, Philips Radiation Oncology Systems, Madison, WI). Two-dimensional anterior DRRs were reconstructed from the CT images at an optimized resolution of 512 × 512 pixels, and source-to-axis distance (SAD) of 100 cm. The heart was highlighted on the DRR based on manual heart contours, to facilitate the placement of NCs during the adaptation process. For retrospective studies on historically treated patients, the heart would also be highlighted on the digitized 2D fluoroscopic planning images. Two-dimensional fluoroscopic images has higher soft tissue constrast than DRRs as they were generally taken at a lower kVp setting (80-100 kVp) at the time of conventional simulation. A graticule was included for image scaling of the DRRs.

### C. Population-to-Patient Specific Deformation Models

Using an in-house FEM-DIR platform, MORFEUS [10,15], a male and a female biomechanical-based finite-element population heart model were constructed from combining manually delineated heart contours on the CT images of 6 male or 6 female HL patients. This process involved 1) exporting heart contours as binary masks from the treatment planning system, 2) summation of the binary mask files using a logical OR function, and 3) conversion of the data into a 3D tetrahedral finite element volume mesh, using the Interactive Data Language software (IDL v6.3, ITT, Boulder, CO). The population models, which encompass all original Base patients' hearts, served as an initial 3D representation of the general shape and volume of the heart.

The male or female population heart model was then deformed into each of the 35 patients (12 Base patients that made up the population models and 23 Reference patients to generate 3D population-to-Reference patient deformation templates). Each deformation was carried out by first deforming the surface nodes of the FEM population heart model onto a surface defined by the secondary Reference heart model. This is followed by deformation of the internal structure of the heart according to the biomechanical properties assigned to the heart model (Young's modulus: 7.8 kPa and Poisson's ratio: 0.45) [15]. Accuracy of each deformation was visually verified using the HyperMesh software (HYPERMESH v 9.0, Altair Engineering, Troy, MI). The 23 resultant population-to-Reference patient deformation template maps were then adapted to rebuild 3D heart models of 15 additional Test patients using only 2D DRRs, as described below.

### D. Identification of Matching Patients

**Figure 2:**
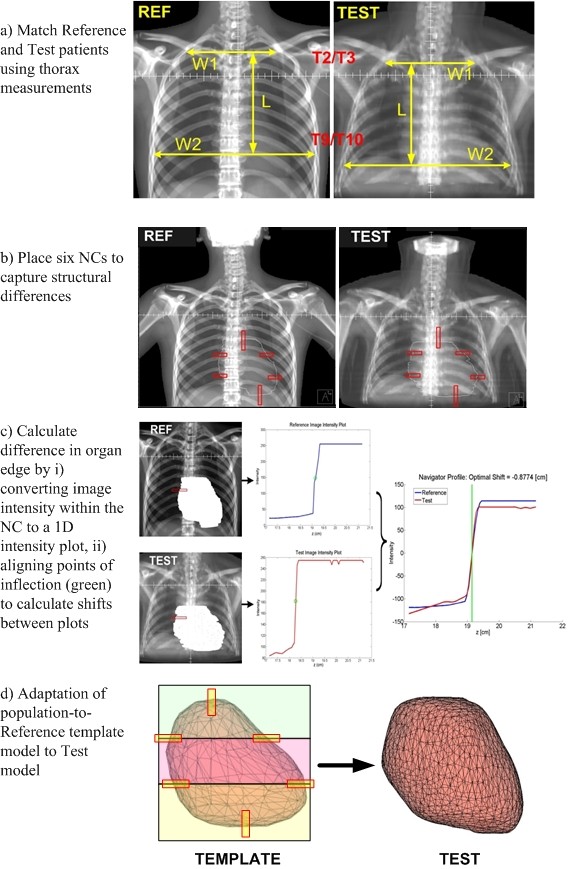
**NC Adaptation Process**. There are four main steps in this process: a) Matching Reference and Test patients using thorax measurements, b) Placing 6 NCs to capture structural differences, c) Calculating the difference in organ edge based on shifts in intensity gradients, d) Adapting population-to-Reference template model to Test model.

To reconstruct the 3D heart models of the Test patients using the population-to-Reference patient specific deformation templates, a similar Reference patient was selected for each Test patient based on thorax measurements. In order to make comparable measurements, the Reference and Test patients' coronal DRRs were scaled to similar magnification by reconstructing the images at 100 cm SAD. Three thorax measurements (W1, W2, L) were acquired on the Reference and Test DRRs (Figure 2a). The width of the superior thorax (W1) and inferior thorax (W2) were measured as the horizontal distance between the inner borders of the ribs with the chest at the vertebral body level of T2/T3 and T9/T10, respectively. The length of the thorax (L) was measured as the vertical distance between the vertebral bodies of T2/T3 and T9/T10. A least-squares difference (LSD) between each Reference and Test patient within the same gender group was calculated based on the three measurements [Equation (1)].

(1) $$LSD=\sqrt{{\left(W1_{test}-W1_{ref}\right)}^{2}+{\left(W2_{test}-W2_{ref}\right)}^{2}+{\left(L_{test}-L_{ref}\right)}^{2}}$$

Matching Reference-Test pairs (RTPs) were identified based on lowest LSD scores (highest similarity) within their own gender group. A total of 15 RTPs were selected for this study. It is important to note that thorax measurements were used to identify patients with similar heart sizes, because radiographic film studies have expressed a strong relationship between thoracic measurements and cardiac sizes [16,17]. In particular, the cardiothoracic ratio has traditionally been used as a basis for distinguishing age groups, ethnic groups, and patients with cardiac abnormality [18-20].

### E. Quantification of Structural Differences

To refine selected Reference patient's deformation template maps for rebuilding Test patients' 3D heart models, the structural and positional differences between each RTP was quantified. The overall quantification process involved the following steps.

First, provided that the Reference and Test DRRs were initially reconstructed at an SAD of 100 cm, the user selected two points that corresponded to 10 scales on the graticule first on the Reference patient image then on the Test patient image. The software then automatically scaled the Test image to correspond to the Reference image (ie: 1 cm on the Test image corresponds to 1 cm on the Reference image).

Second, the anterior DRRs of the Reference and Test patients were rigidly aligned using the mid sternal notch (T4/T5), and the mid spinous processes of T6 and T8. This places the two images in the same comparable spatial location. The user then sets the *x *and *y *coordinates of the crosshair on the Reference image to the *x *and *y *coordinates of the isocenter on the Reference CT image as defined in the treatment plan. This provides a positional linkage between the 2D image and the 3D population-to-Reference patient deformation map.

Third, NCs were applied onto the two images. A NC was first placed on the Reference DRR by the user, then a spatially corresponding NC was automatically placed on the Test DRR by the NC software. A NC is a rectangular region of interest placed on two images that captures the intensity value differences within the region and generates an image intensity plot [13]. The locations for placing the NCs were selected based on clearly defined boundaries of the heart as shown on the Reference and Test patient's radiograph (Figure 2b). To account for differences in the superior and inferior edge of the heart, a NC was placed superiorly and inferiorly. For Test cases where the inferior edge of the heart is difficult to outline on the 2D image as a result of inherent limitations of using a 2D radiographic film, the dome of the diaphragm was used to define that inferior edge. For Test cases where the superior edge is difficult to define due to blurring of adjacent structures with similar tissue densities (aorta, superior vena cava, pulmonary veins and arteries), the inferior edge of the carina, which is at the anatomical level of the pulmonary artery (located bilaterally and carries deoxygenated blood from the heart to the lungs) was used to define the superior edge of the heart. To account for lateral edge differences between Reference and individual Test patient's heart, locations where there is a significant change in curvatures between the Test and Reference patient heart structure were selected. Four NCs were generally placed at the major circumflexes of the heart, one at the edge of each of the four heart chambers (Right/Left atrium, Right/Left ventricles). In total, six NCs were placed at the edges of the heart: 2 vertical NCs (0.5 × 4.0 cm) for quantifying edge differences in the superior and inferior directions, 4 horizontal NCs (2.0 × 0.5 cm) for quantifying edge differences in the left and right directions at the 4 major circumflexes of the heart (Figure 2b).

Fourth, the image intensity within each NC was converted into a 1D intensity plot function. Finally, using a second derivative calculation, the difference or shift in intensity plots of each pair of NCs were calculated based on the differences of their points of inflection (POI) [13,14]. POI occurs where the rate of change in the intensity values is the greatest, thus indicating the edge of the organ. Figure 2c illustrates this process for one of the NCs in the superior medial edge position.

### F. Adaptation of Population Heart Model

**Figure 3:**
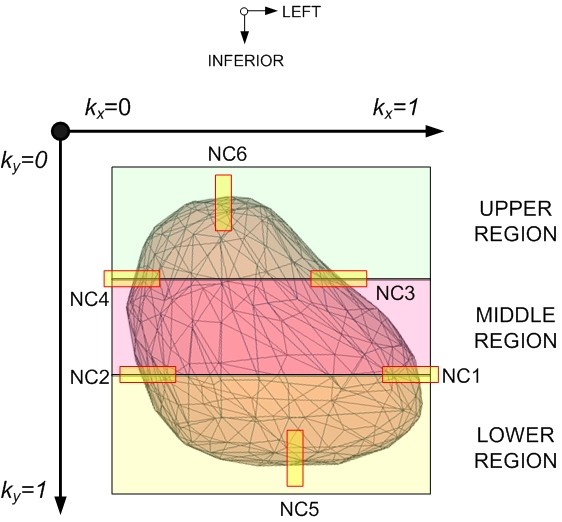
**Adaptation of population-to-Reference template heart model using linear and bilinear interpolation methods**. NC5 and NC6 captured the inferior and superior displacements between the Reference and Test volumes [Eq.(1)]. The heart was further divided into upper, middle and lower regions using NC1, NC2, NC3, and NC4. The lateral adjustment of a node in the lower and upper region was determined by NC1, 2 [Eq.(4)] and NC3, 4 [Eq.(2)], respectively, using the linear method. The adjustment of a node in the middle region was calculated from all four NCs using the bilinear method [Eq.(3)].

To generate population-to-Test patient specific deformation maps from the population-to-Reference deformation template maps, a series of linear and bilinear adaptation calculations were performed using the detected 1D edge differences [13,21]. As shown on Figure 2d, the four horizontal NCs divided the organ into three regions (upper, middle and lower) in order to facilitate the adaptation process as described below. The deformation at each node of the template model was adjusted longitudinally [Eq.(1)] and laterally [Equation (2), (3), or (4)] based on the node's relative position to the NC locations placed on the heart. Figure 3 shows the application of the 4 equations and derivation of each equation is described as below.

#### Longitudinal Displacements

Equation 2 is a linearly weighted equation that was derived from the linear interpolation of the longitudinal shifts at the two known navigator channel positions, NC_5 _and NC_6_. Based on the longitudinal shifts detected by NC_5 _and NC_6_, this equation refined and updated the position of each node in the population-Reference heart model Δ(*y_0_*) to become Test patient specific ∂(*y_0_*).

(2) $$\partial \left(y_{0}\right)=\Delta \left(y_{0}\right)\left[\left(\frac{\Delta NC_{5}+\partial NC_{5}}{\Delta NC_{5}}\right)\left(k_{yo}\right)+\left(\frac{\Delta NC_{6}+\partial NC_{6}}{\Delta NC_{6}}\right)\left(1-k_{y0}\right)\right]$$

The longitudinal adjustment of a node displacement (*y_0_*) from population-to-Test patient specific, ∂(*y_0_*), is equal to the predicted population-to-Reference patient deformation at the same node, Δ(*y_0_*). This adjustment is refined by a linear weighting of the difference in the heart edges between the Reference and Test patient DRRs determined at each NC, ∂*NC_5 _*(superior edge), and ∂*NC_6_*, (inferior edge), against the predicted displacement of the population-to-Reference patient deformation map at the NC node positions, Δ*NC_5 _*and Δ*NC_6_*, respectively. The adjustment is further weighted by the relative distance of the adapting node to the two NC positions, *k_y _*and (1-*k_y_*), where *k_y _*varies between 0 and 1 (0 is most superior and 1 is most inferior edge of the heart).

#### Lateral Displacements

Equations (3), (4), and (5) account for the lateral adjustments of the nodes within the upper, middle, and lower regions of the heart, respectively. Four NCs were used because they help quantify major lateral edge differences of the four heart chambers (atrium & ventricles). At the same time, these 4 NCs provided a division of the heart that allowed for more accurate nodal adjustments, by deforming each node based on the NCs that were closest to it. Equations 3 and 5 are linearly weighted equations that were derived from the linear interpolation of the shifts calculated at its respective navigator channel positions. The position of the nodes in the upper region (Eq. 3) was calculated based on its relative position to NC_3 _and NC_4. _The position of the nodes in the lower region (Eq. 5) was calculated based on its relative position to NC_1 _and NC_2. _Based on the lateral shifts detected by its respective NC positions, equation 3 and 5 updates the position of each node in the population-Reference heart model Δ(y_0_) to become Test patient specific ∂(y_0_). Equation 4 is a bilinearly weighted equation that is derived from bilinear interpolating the shifts calculated at the four lateral NC positions, NC_1_, NC_2_, NC_3_, and NC_4_. Unlike linear interpolation, bilinear interpolation considers the closest 2 × 2 neighboring NC positions surrounding the unknown nodal position, Δ(x_0_). It then takes a weighted average of these four positions to arrive at its final, interpolated value, ∂(x_0_). The weight on each of the 4 NC positions is based on the computed nodal position (in 2D space) from each of the known NC positions. A bilinear equation is necessary, because it provides a more accurate deformation of the population-Reference model by accounting all possible shifts as a result of the differences detected by the four navigator channels.

#### Upper Region

(3) $$\partial \left(x_{0}\right)=\Delta \left(x_{0}\right)\left[\left(\frac{\Delta NC_{3}+\partial NC_{3}}{\Delta NC_{3}}\right)\left(k_{x_{0}}\right)+\left(\frac{\Delta NC_{4}+\partial NC_{4}}{\Delta NC_{4}}\right)\left(1-k_{x_{0}}\right)\right]$$

In Equation (3), the lateral adjustment of a node displacement from population-to-Test patient specific, ∂(*x_0_*), is equal to the predicted population-to-Reference patient displacement, Δ(*x_0_*). This is adjusted by a linear weighting of the shifts calculated at NC_3_, ∂*NC_3_*, and NC_4_, ∂*NC_4_*, against the predicted population-to-Reference patient deformation at the NC_3 _and NC_4 _positions, Δ*NC_3 _*and Δ*NC_4_*. The difference weighting was further adjusted by the relative distance of the adapting node to the two NC positions, *k_x _*and (1- *k_x_*), where *k_x _*varies between 0 and 1 (0 is the most right and 1 is the most left edge of the heart).

#### Middle Region

(4) $$\partial \left(x_{0}\right)=\Delta \left(x_{0}\right)\left[\begin{array}{c} \left(\frac{\Delta NC_{1}+\partial NC_{1}}{\Delta NC_{1}}\right)\left(k_{x_{0}}\right)\left(k_{y_{0}}\right)+\left(\frac{\Delta NC_{2}+\partial NC_{2}}{\Delta NC_{2}}\right)\left(1-{k_{x}}_{{}_{0}}\right)\left(K_{y_{0}}\right)\\ +\left(\frac{\Delta NC_{3}+\partial NC_{3}}{\Delta NC_{3}}\right)\left(k_{x_{0}}\right)\left(1-{k_{y}}_{{}_{0}}\right)+\left(\frac{\Delta NC_{4}+\partial NC_{4}}{\Delta NC_{4}}\right)\left(1-{k_{x}}_{{}_{0}}\right)\left(1-{k_{y}}_{{}_{0}}\right) \end{array}\right]$$

In Equation (4), the deformation at a node, ∂(*x_0_*), is equal to the population-to-Reference patient predicted deformation, Δ(*x_0_*). This calculation is adjusted using bilinear interpolation of the edge differences between the Reference and Test DRRs, determined at the four NC positions, ∂*NC_1_*, ∂*NC_2_*, ∂*NC_3_*, and ∂*NC_4_*, against the predicted displacement of the population-to-Reference patient deformation map at the NC node positions, Δ*NC_1_*, Δ*NC_2_*, Δ*NC_3_*, and Δ*NC_4_*, respectively. The shift weighting is determined by the relative distance of the node to the four NC positions, *k_x_*, *k_y_*, (1- *k_x_*), and (1- *k_x_*) (where 0 is the most right and superior edge of heart).

#### Lower Region

(5) $$\partial \left(x_{0}\right)=\Delta \left(x_{0}\right)\left[\left(\frac{\Delta NC_{1}+\partial NC_{1}}{\Delta NC_{1}}\right)\left(k_{x_{0}}\right)+\left(\frac{\Delta NC_{2}+\partial NC_{2}}{\Delta NC_{2}}\right)\left(1-{k_{x}}_{0}\right)\right]$$

Equation (5) follows the same logic as Equation (3), where NC_1 _and NC_2 _represent the right and left positions, respectively.

Based on these equations, the dense population-to-Reference deformation field, which represents all the nodal coordinates of the Reference heart model was modified according to the position of each node relative to each NC location in order to calculate the deformation from population-to-Test patient. To ensure continuity, the interpolated displacements were then applied to each of the nodal points that make up the finite-element based population-Reference deformation heart model to reconstruct Test-patient-specific 3D heart models (Figure 2d).

### G. Accuracy of Adapted Heart Models

The accuracy of each NC-adapted model (V_1_) in representing the actual 3D Test volume (V_2_) was evaluated by calculating the percentage volume overlap between them using the Dice coefficient as shown in Equation (6) [22,23]. In order to compare the NC-adapted model and actual Test model volumetrically, the two volumes were first rigidly aligned by their center of mass. The Test volume (V_2_) was created from the 3D heart contours generated from planning CT.

(6) $$Dice=\frac{2\left(V_{1}\cap V_{2}\right)}{V_{1}+V_{2}}\times 100$$

The Dice coefficient was chosen in favor of simple matching similarity measure, because it does not "over-estimate" the percentage overlap of false-positive volumes [22]. For comparison, the Dice coefficient expressed as a percentage between the original Reference (without NC adaptation) and its corresponding Test volume was calculated. Results with and without NC adaptation were compared using a Student's paired T-test.

To provide context for the accuracy of the reconstruction method, the Dice results were compared to the heart volume variation due to free-breathing lungs and diaphragm motion. 4D-CT images of 9 randomly selected lung cancer patients were analyzed. These CT images were acquired during synchronized respiration, in cine-mode, using a four-slice fan-beam CT scanner (Discovery LS, GE, Waukesha, WI). Sets of end-inhale and exhale images were transferred to the treatment planning system to assess the maximum variation in heart volume and position. Heart contours generated from both image sets were exported from the treatment plan as binary mask files and used to calculate their percentage volume overlap using the Dice coefficient (V_1 _= Inhale volume, V_2 _= Exhale volume). A paired T-test was performed between the NC-adapted and respiratory heart Dice results. This comparison was made, because the largest displacement occurs in the superior-inferior direction, as a result of free-breathing respiratory motion during radiotherapy [24]. It is important to note that a rigid registration of the inhale and exhale volumes was not performed here because it is the intention of this study to determine how the accuracy of the NC reconstruction technique compare to the inherent positional and volumetric heart variation due to respiratory motion.

## Results

### A. Population Deformations

**Figure 4:**
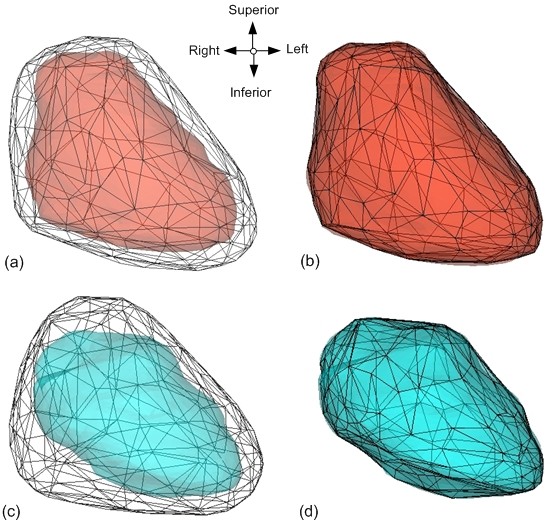
**Visual inspections of the deformation between population and one of the Base or Reference models**. The population model (black) is shown in mesh form, and the Base (red) or Reference (blue) models are shown as solid forms. Figures 4a and 4c represent each model before the deformation, while figures 4b and 4c represent each model after the deformation.

Figures 4a and 4b display the graphical accuracy of the population model (mesh) and one of the Base patients' model (solid) before and after the deformation, respectively. Figures 4c and 4d verify the surface deformation between the same population model (mesh) and one of the Reference patient's heart models (solid). Qualitative assessment of the deformation between the population and each of the Base or Reference heart models shows good volumetric and spatial agreements.

### B. Identification of Matching Reference-Test Pairs

Table 1 summarizes the three thorax measurements and LSD scores of the 15 RTPs (7 male pairs, 8 female pairs). Male subjects are denoted by the "M" identifier and female subjects are denoted by the "F" identifier. The Reference subjects were selected within the same gender group as the Test subjects. In addition, these five Reference patients were chosen, because they produced the lowest LSD scores (highest similarity) with the Test subjects. The LSD scores of the 15 RTPs ranged from 0.4 to 1.2 cm.

**Table 1:** Summary of LSD scores for the 15 RTPs

	Reference Subjects			Test Subjects			
	
RTP	W1	W2	L	W1	W2	L	LSD
M1	15.0	24.8	14.3	14.7	25.3	14.4	0.59
M2	15.0	24.8	14.3	15.6	24.9	14.4	0.62
M3	15.0	24.8	14.3	15.2	25.2	14.6	0.54
M4	15.0	24.8	14.3	15.3	25.0	14.0	0.47
M5	15.0	24.8	14.3	14.6	24.9	13.8	0.65
M6	15.1	27.2	16.5	15.3	27	15.6	0.94
M7	15.1	27.2	16.5	14.1	26.7	16.2	1.16
F1	14.6	24.9	13.8	15.1	24.1	13.9	0.95
F2	14.6	24.9	13.8	14.7	25.3	14.4	0.73
F3	14.6	24.9	13.8	14.9	24.0	13.5	0.99
F4	14	26.1	16	14.1	26.7	16.2	0.64
F5	14	26.1	16	14.7	26	16.2	0.73
F6	14	26.1	16	14.3	26.9	15.3	1.10
F7	15.1	27.2	16.5	15.8	26.8	16.9	0.90
F8	15.1	27.2	16.5	15	27.2	15.7	0.81

W1, W2 represent the thorax width measured at T2/T3 and T9/T10, respectively. L represents the length of thorax measured from T2/T3 to T9/T10.

### C. Accuracy of Adapted Heart Models

**Figure 5:**
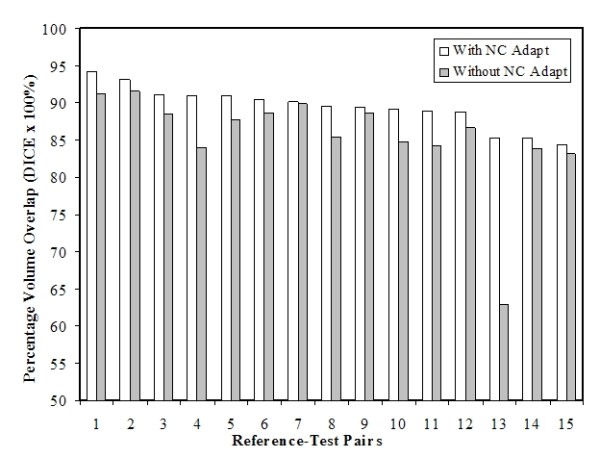
**Distribution of percentage volume overlaps with and without NC adaptation**. The original Reference (blue) and NC-adapted Reference (black) heart models are shown in mesh form. The actual Test heart model is shown in red solid form. Ideally, the NC-adapted Reference and Test model should match.

**Figure 6:**
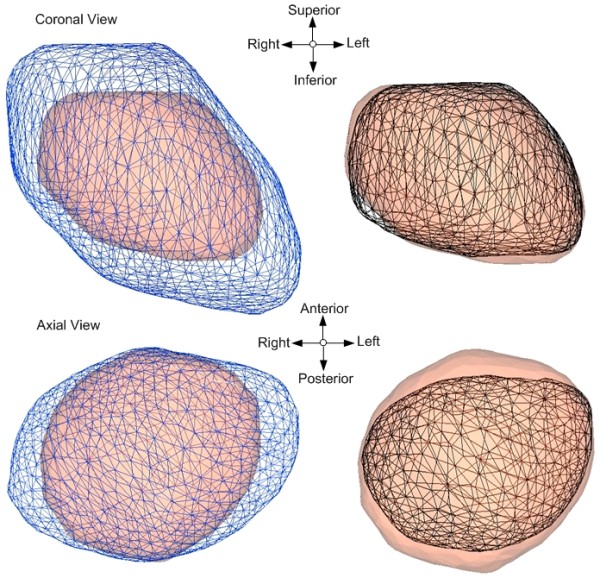
**Graphical representation of original Reference, NC-adapted Reference and actual Test model for Test patient 13**. The original Reference (blue) and NC-adapted Reference (black) heart models are shown in mesh form. The actual Test heart model is shown in red solid form. The Reference patient's heart is more elongated than the Test patient's heart volume.

Table 2 summarizes the Dice results between 1) the population-to-Reference patient deformation models and the actual Test models (Without NC adaptation), 2) the NC-adapted models and the actual Test models (With NC adaptation), and 3) the free-breathing inhale and exhale heart models (Baseline). On average, the percentage volume overlap, as measured by the Dice coefficient is greater with (89.4 ± 2.8%) than without (85.4 ± 6.8%) NC adaptation. A paired T-test shows the NC adaptation process made significant improvements to the population-to-Reference patient deformation models (p = 0.01). Figure 5 shows the distribution of percentage overlap with and without NC adaptation for the 15 RTPs. It is important to note that the percentage volume overlap with and without NC adaptation appears to be larger for case 13, because the Reference patient chosen for Test 13 seems to have a more elongated heart volume than the Test patient (Figure 6). Excluding this case will not significantly change the results of this study, as the recalculated Dice coefficient between NC-adapted and actual Test model is 89.7 ± 2.6%, and the recalculated p-value remains < 0.01. As predicted, the Dice percentage was higher for all RTPs when NC adaptation was applied. Table 2 also shows that the accuracy of the NC adaptation process, as measured by the Dice coefficient between the NC-adapted model and the actual Test model (Average Dice = 89.4 ± 2.8%) is in the same order of variation seen in free-breathing heart volumes (Average Dice = 90.1 ± 3.3%). A paired T-test shows that the Dice results with NC-adaptation are comparable to those between free-breathing heart models (p = 0.62), indicating that the inaccuracy in volumes reconstructed with the NC adaptation process are within the limitations of heart motion uncertainty due to normal respiration during radiotherapy treatment.

**Table 2:** Summary of percentage volume overlap or Dice results

Volume Overlap Tests *		**Ave**.	**Min**.	**Max**.	SD
1	Ref. (Not NC Adapted) & Test	85.4	62.9	91.6	6.8
2	NC-adapted & Test	89.4	84.4	94.2	2.8
3	FB inhale & exhale subset	90.1	86.6	94.1	3.3

* Dice was calculated between (1) the Reference patient template model and the actual Test patient heart model; (2) the NC-adapted heart model and the actual Test patient heart model; and (3) the subset Free-breathing (FB) patient CT contour volumes at exhale and inhale.

**Figure 7:**
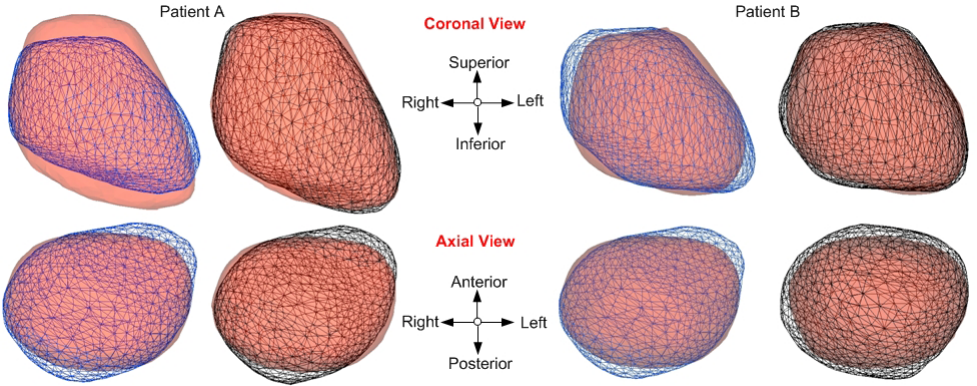
**Graphical representation of original Reference, NC-adapted Reference and actual Test models for two patients (A, B)**.

Figure 7 is a graphical representation of two RTPs [Patient A (RTP 5): Dice = 91.0% (NC adapted) vs. 84.0% (Not adapted) and Patient B (RTP) 10: Dice = 91.3% (NC adapted) vs. 88.2% (Note adapted)], where the population-to-Reference patient specific model (before adaptation) is represented in blue mesh, the NC-adapted Reference patient model (after adaptation) is shown in black mesh, and the actual Test patient model is shown in solid red. Volumetric comparison shows that NC adaptation was most prominent in the left-right and inferior-superior directions, as indicated by the surface improvements in the black mesh and red solid models. Due to the lack of a lateral film, regions of residual non-overlapping volumes are most noticeable in the anterior-posterior direction.

## Discussion

The reconstructed 3D heart volumes using the advanced NC adaptation process match well with the volumes obtained from CT. The Dice results demonstrate that the heart volume can be reconstructed with an accuracy of about 90%, which is comparable to the volumetric and/or positional variation due to respiration (*p *= 0.62). Statistically, it has been shown that the NCs made a significant improvement to the population deformation models (*p *= 0.01).

In this study, 3D heart volumes were reconstructed without lateral depth information. Although a "similar" Reference patient was identified (by thorax measurements) to approximate the lateral depth of the Test patient's heart, there remains an observable discrepancy in the anterior-posterior direction of the reconstructed models. This difference is largely due to the limitation that only anterior-posterior simulator films are available from historical 2D treatment plans. This limitation could introduce error in the computation of heart doses, as variations in heart-tissue density may cause the primary photons to become more or less attenuated than expected. However, current study results also indicated that this reconstruction error is no greater than the uncertainty imposed by a patient's free-breathing motion. Additionally, variation in day-to-day patient positioning will affect dose actually delivered to the heart during treatment [25,26]. A previous study on the reproducibility of treatment setup for mantle irradiation in HL patients, using sequential port films, found that 29% of the films had minor deviations from original simulation film and 5% were considered unacceptable [26]. Errors were noted in the superior and inferior mediastinum and could only be eliminated by resimulation [26]. Taken together, we believe that the inaccuracies of the reconstruction technique described here are no greater than that caused by organ motion due to free-breathing and other setup uncertainties that are part of clinical practice.

The current NC adaptation technique is entirely based on soft-tissue alignment to allow for complete heart deformation. The manual contours of the heart were included on the DRR to facilitate NC placement. For retrospective studies, the same contours can be used for defining the heart on the Reference DRR. On digitized 2D fluoroscopic films of retrospective patients, the heart shape could also be highlighted as the soft tissue heart contour would in fact be better enhanced by the use of lower kVp at the time of conventional simulation. To further enhance tissue contrast on a 2D simulation film, a high-resolution diagnostic film scanner, combined with film processing software with an adjustable grayscale feature could be used. While it is recognized that overlapping boundaries (heart & ribs) may sometimes be difficult to define on radiographic films, the NC technique overcome this limitation by utilizing only sections of the organ edge in the adaption process. This is one of the major advantages of the NC technique over common segmentation techniques, which require complete organ boundary definition [27,28]. Specifically, six NCs were applied only to sections of the edges that would most significantly change the shape of the heart (superiorly, inferiorly, & circumflexes of the four chambers) and the entire heart volume was quickly regenerated using the adaptation process.

Another key advantage of the advanced NC technique is its ability to delineate organ shape curvatures using a combination of linear and bilinear adaptation. This technique incorporates a bilinear method to allow multiple NCs to detect shape differences along the same organ edge. Potentially, cubic or bicubic interpolation methods could be used with multiple NCs to further improve reconstruction accuracy [21,29]. However, the number of NCs employed should be organ-dependent, and calculation times should be balanced with accuracy outcomes. For example, heart volume overlap was computed using two additional NCs in the superior and inferior direction. The additional NCs did not result in a significant increase in volumetric accuracy.

The capacity to recreate 3D organ volumes from 2D planning data potentially allows for better correlation between organ dose and late onset toxicity. Investigation is underway to 1) reconstruct the 3D CT volumes (lungs & heart) and treatment plans of current patients for dosimetric validation of the current adaptation technique, and 2) reproduce the 3D dosimetry of retrospective 2D treatment plans in order to assess and refine existing dose, volume, and outcome relationships [4-7]. The latter process involves combining the use of the traditional film digitizer (ADAC Numonics-Accugrid Digitizer) with the 3D CT-based treatment planning system in order to incorporate shielding (lungs, heart, larynx, C-spine, humeral heads) from a 2D radiograph onto the adapted CT image.

The current development is a preliminary study, limited to the reconstruction of the overall heart volume. While this technique can offer a general dose-volume relationship of the heart, there remains several constraints when combined with 3D dosimetry data. First, localized radiation effects of specific cardiac structures remain unknown. Existing models tend to discard organ-specific spatial information, assuming all regions of the heart are of equal radiosensitivity [4-7]. However, this assumption is inaccurate, as the heart can be defined both anatomically (cardiac chambers, pericardium) and physiologically (cardiac cycles) [7,30]. Second, the long term cardiac effects of radiation may be confounded with other treatment factors that may require further investigation. For instance, adjuvant chemotherapy is increasingly common for treatment of HL. Patients treated with chemotherapy (anthracyclines) alone were found to have increased cardiac toxicity [31,32]. In contrast, non-chemo lipid-lowering treatments (statin) have demonstrated risk reduction in cardiac death [33,34]. Therefore, in order to provide more accurate representation of the organ, future studies could aim at localization of the sub-regions of the heart, modeling the interaction of these sub-regions, and investigation of the combined late effects of radiation and other treatment regimens.

## Conclusions

In summary, an advanced NC adaptation technique to reconstruct 3D heart volumes from 2D planning images has been developed. The reconstruction accuracy is comparable to the uncertainties observed in respiratory motion and thus would not be the limiting factor in estimating heart tissue exposure. A better understanding of the relationships between dose, volume and cardiac toxicity from conventional treatments can provide means for evaluation and refinement of existing dose-tissue constraints and NTCP models, in order to make improvements to modern radiotherapy techniques.

## Abbreviations

1D: One dimensional; 2D: Two dimensional; 3D: Three dimensional; CT: Computed tomography; DIR: Deformable image registration; DRR: Digitally reconstructed radiograph; FEM: Finite-element based model; HL: Hodgkin's lymphoma; LSD: Least squares difference; NC: Navigator channel; NTCP: Normal-tissue complication probability; POI: Point of inflection; RT: Radiation therapy; RTP: Reference-Test pairs; SAD: Source-to-axis distance; T2/T3: Inter-vertebral space between second and third thoracic vertebrae; T9/T10: Inter-vertebral space between ninth and tenth thoracic vertebrae.

## Competing interests

The authors declare that they have no competing interests.

## Authors' contributions

AN carried out the 3D heart reconstruction study, participated in the design of the Navigator Channel adaptation process, acquired data, performed statistical analysis, drafted and revised the manuscript. TNN participated in the design of the Navigator Channel adaptation process, contributed to data analysis and interpretation, and helped to draft the manuscript. JLM participated in the design of the Navigator Channel adaptation process, contributed to data analysis and interpretation, and helped revise the manuscript. DCH conceived of the study, provided the financial support, participated in its design and coordination, contributed to data analysis and interpretation, and helped revise the manuscript. MBS participated in the design of the Navigator Channel adaptation process, contributed to data analysis and interpretation, and helped revise the manuscript. KKB provided study materials, financial support, participated in its design and coordination, contributed to data analysis and interpretation and helped revised the manuscript. All authors read and approved the final manuscript.

## Pre-publication history

The pre-publication history for this paper can be accessed here:

http://www.biomedcentral.com/1756-6649/12/1/prepub
